# Mental disorders and the risk for the subsequent first suicide attempt: results of a community study on adolescents and young adults

**DOI:** 10.1007/s00787-017-1060-5

**Published:** 2017-10-11

**Authors:** Marcel Miché, Patrizia Denise Hofer, Catharina Voss, Andrea Hans Meyer, Andrew Thomas Gloster, Katja Beesdo-Baum, Roselind Lieb

**Affiliations:** 1 Division of Clinical Psychology and Epidemiology, Department of Psychology, University of Basel, Missionsstrasse 60/62, 4055 Basel, Switzerland; 2 Behavioral Epidemiology, Technische Universität Dresden, Dresden, Germany; 3 Max Planck Institute of Psychiatry, Munich, Germany; 4 Institute of Clinical Psychology and Psychotherapy, Technische Universität Dresden, Dresden, Germany; 5 Division of Clinical Psychology and Intervention Science, Department of Psychology, University of Basel, Basel, Switzerland

**Keywords:** First suicide attempt, Adolescents and young adults, Mental disorders, Community sample, Onset, Prospective design

## Abstract

Adolescents and young adults represent the high-risk group for first onset of both DSM-IV mental disorders and lifetime suicide attempt (SA). Yet few studies have evaluated the temporal association of prior mental disorders and subsequent first SA in a young community sample. We examined (a) such associations using a broad range of specific DSM-IV mental disorders, (b) the risk of experiencing the outcome due to prior comorbidity, and (c) the proportion of SAs that could be attributed to prior disorders. During a 10-year prospective study, data were gathered from 3021 community subjects, 14–24 years of age at baseline. DSM-IV disorders and SA were assessed with the Munich-Composite International Diagnostic Interview. Cox models with time-dependent covariates were used to estimate the temporal associations of prior mental disorders with subsequent first SA. Most prior mental disorders showed elevated risk for subsequent first SA. Highest risks were associated with posttraumatic stress disorder (PTSD), dysthymia, and nicotine dependence. Comorbidity elevated the risk for subsequent first SA, and the more disorders a subject had, the higher the risk for first SA. More than 90% of SAs in the exposed group could be attributed to PTSD, and over 30% of SAs in the total sample could be attributed to specific phobia. Several DSM-IV disorders increase the risk for first SA in adolescents and young adults. Several promising early intervention targets were observed, e.g., specific phobia, nicotine dependence, dysthymia, and whether a young person is burdened with comorbid mental disorders.

## Introduction

Adolescents and young adults are the age group at highest risk for the first onset of commonly occurring mental disorders [1, 2]. This life stage has also been identified as a critical period for the onset of the first suicide attempt (SA) [for reviews, see 3, 4].

Many studies have examined associations between mental disorders and SAs among adolescents and young adults [e.g., 5, 6–11]. Almost all the reported associations, however, are of a cross-sectional nature. As such, they provide no information on the temporal effects.

To date, only a handful of population-based studies have taken the temporal sequence into consideration, that is, whether temporally prior mental disorders increase the risk for subsequent first SA. Using different methodologies, these studies generally [12–17] although not always [18–20] found evidence that mental disorders occurring in adolescence and young adulthood predict the subsequent first onset of SA. Yet most of these studies focused either on disorder groups (e.g., mood, anxiety, or substance use disorders; see Fergusson et al. [12]) or on a few individual predictor disorders (for nicotine dependence, see Bronisch et al. [15]; for phobia, general anxiety disorder (GAD), and panic, see Boden et al. [13]; for major depressive disorder, see Lewinsohn et al. [14]). Drawing comparative conclusions is thus difficult because on the one hand, disorder groups do not reveal the contributions of the individual disorders to the reported risk estimates. On the other hand, studies that assessed one or a few individual disorders cannot reveal the risk pattern of a wide range of disorders.

We identified two cross-sectional population-based studies that simultaneously examined for a wide range of mental disorders whether retrospectively assessed prior mental disorders increase the risk for subsequent first onset of SA among youth [16, 17]. Borges et al. [16] used data of the Mexican Adolescent Mental Health Survey (MAMHS), a representative survey of 3005 adolescents aged 12–17 years in Mexico City. For DSM-IV [21] anxiety, mood, and substance use disorders, this group found that with the exception of panic disorder, posttraumatic stress disorder (PTSD), and alcohol abuse, each of the assessed mental disorders (i.e., GAD, specific phobia, social phobia, alcohol dependence, drug abuse) increased the risk for subsequent first SA. In the second study, Nock et al. [17] evaluated associations between prior DSM-IV mental disorders and subsequent first SA on the basis of the U.S. National Comorbidity Survey Replication Adolescent Supplement (NCS-A) in which a total of more than 6000 adolescents 13–18 years of age were assessed. Adjusting for comorbidity, Nock et al. [17] replicated the finding that major depression/dysthymia and any bipolar disorder and in addition any eating disorder predicted subsequent SA. Also consistent with the findings of Borges et al. [16], prior panic disorder and alcohol abuse were not associated with subsequent SA. In contrast to the findings of the Mexican survey, Nock et al.’s [17] findings showed no associations between prior specific phobia, social phobia, or GAD with subsequent SA.

To date, there is almost no information on the proportion of SAs among young people that is attributable to prior mental disorders in the general population. Such information is the basis of any informed prevention effort made on the population level. Only Boden et al.’s [13] longitudinal Christchurch Health and Development Study (CHDS) of over 1000 adolescents and young adults estimated population attributable fractions and found that the presence of any anxiety disorder accounted for 7.5% of the SAs in the cohort. So far, comparable information has never been reported for other mental disorders in the younger general population.

Against this background, we want to extend our own and others’ earlier research on the associations between prior mental disorders and subsequent first onset of SA by extending the observed age period to age 34 years and, additionally, estimating for adolescents and young adults fractions of SAs attributable to specific mental disorders.

Using data that were collected prospectively during the 10-year follow-up of the early developmental stages of psychopathology (EDSP) study,

1. we estimated the overall association of a wide range of DSM-IV mental disorders with the risk for subsequent first SA;
2. we evaluated temporal associations between the number of prior mental disorders and subsequent first SA;
3. and we estimated the proportion of first SAs for the specific disorder groups and the total population that could conceivably be prevented by effective prevention in the first three decades of life.

## Methods

### Design and sample

The EDSP study has a 10-year prospective-longitudinal design. It assesses DSM-IV mental disorders and associated risk factors in a community sample. A sample of 3021 subjects (aged 14–24 years) was first assessed (T0) in 1994, followed by three additional assessments until 2005 (T1, T2, and T3). Subjects were selected from government registries of the greater Munich area, Germany. The focus of the study on the early development of psychopathology is expressed by the sampling scheme: compared to 16- to 21-year-old individuals, those who were 14–15 years old were sampled at twice the probability, whereas 22- to 24-year-old individuals were sampled at half the probability. Subsequent analyses took this scheme into account using sample weights. At baseline, the response (participation) was 70.9% (*N* = 3021). T1’s response (range 1.2–2.1 years after baseline) was 88% (*N* = 1228), and only those subjects aged 14–17 years at baseline were assessed. At T2 (range 2.8–4.1 years after baseline) 2548 subjects were interviewed (response 84.3%); at T3 (range 7.3–10.6 years after baseline) 2210 were interviewed (response 73.2%). At baseline, most of the subjects were attending school (51.8%) and were living with their parents (72.7%). The majority were classified as belonging to the middle class (95.9%). More detailed information on methods, design, and sample characteristics has been presented elsewhere [2, 22, 23]. The EDSP project was approved by the Ethics Committee of the Medical Faculty of the Dresden University of Technology. All subjects provided informed consent.

### Diagnostic assessment of DSM-IV mental disorders

All DSM-IV mental disorders were assessed by face-to-face interviews, using the computer-assisted Munich-Composite International Diagnostic Interview (DIA-X/M-CIDI) [24]. The DIA-X/M-CIDI was constructed for the standardized assessment of symptoms, syndromes, and diagnoses of DSM-IV disorders, along with information on age of onset (AOO), duration, and severity. Clinical interviewers, who were extensively trained in using the DIA-X/M-CIDI, interviewed the subjects for 2–3 h. At baseline, the DIA-X/M-CIDI lifetime version was used. At each of the three follow-up assessments, the DIA-X/M-CIDI was modified to obtain information about the period between the last and the current assessment. The DSM-IV disorders were obtained using DIA-X/M-CIDI algorithms. Test–retest reliability and validity for the full DIA-X/M-CIDI have been reported elsewhere [25, 26]. Age, sex, and socioeconomic class were assessed in the sociodemographic section of the DIA-X/M-CIDI. Any childhood/adolescence separation event (i.e., death of mother, death of father, separation/divorce of parents) including AOO was assessed in the family history section of the DIA-X/M-CIDI at baseline.

### Assessment of SAs

SAs were assessed in the depression section of the DIA-X/M-CIDI with the question “Have you ever attempted suicide?” At baseline and T1, only the individuals who acknowledged having had a period of at least 2 weeks with a continuously depressed mood, low energy, or loss of interest [these are the stem questions for major depressive disorder (MDD)] were asked this question. At T2 and T3, a modification was introduced in the depression section to ensure that all individuals were asked questions regarding suicidality in their lifetime, irrespective of whether the stem questions for MDD were confirmed or denied. All subjects who reported that they had attempted suicide were additionally asked for their age at their first SA.

### Data analysis

Data analyses were based on *N* = 3021 subjects. We used the LOCF (last observation carried forward) method, which allowed us to use information from both subjects who dropped out over the study course after baseline and subjects who responded to the follow-up assessments, i.e., for every subject we used the information obtained before dropout, regardless of when it occurred.

Data were weighted by sex, age group, and geographic location at baseline in order to be representative of the original sampling frame. Analyses were performed using R 3.3.3 [27], including the survey [28], survival [29], and ggplot2 [30] packages.

As predictors we used the following DSM-IV diagnoses: panic disorder, agoraphobia with and without panic disorder, social phobia, specific phobia, GAD, PTSD, obsessive compulsive disorder (OCD), major depression, dysthymia, any bipolar disorder, nicotine dependence, alcohol use disorder (alcohol abuse or dependence), drug use disorder (drug abuse or dependence), pain disorder, and any eating disorder. As outcome we used the first SA.

We examined the temporal priority between mental disorders and SAs on the basis of AOO information for both mental disorders and the first SA. Associations between temporally prior mental disorders and temporally subsequent onset of the first SA were estimated using the Cox regression model with person-years as the unit of analysis. Whenever a subject’s reported AOO of a respective mental disorder was equal to the AOO of the first SA, the predictor value in the model was set to 0, thereby favoring conservative results. For each disorder, we fitted a separate Cox model. Each model was adjusted for sex, age group, and socioeconomic class, as well as any other mental disorder (yes/no) and any childhood/adolescence separation event (yes/no), respectively, occurring prior to the predictor disorder. Any other mental disorder and any childhood/adolescence separation event were constructed as proposed by Höfler et al. [31]: For subjects with the predictor disorder, we examined whether the covariate occurred prior to the predictor disorder based on reported AOO. For subjects without the predictor disorder, we proceeded in the same way. However, as for these subjects an AOO does not exist, we estimated the AOO by computing the median of the age at which the predictor disorder “typically begins” (based on all data for which AOO was available). The median age of the predictor disorder was thereby computed separately for the age groups 14–15, 16–17, 18–19, 20–21, and 22–24 years at baseline in order to obtain more precise estimates, since median AOO differed among age groups (results available on request). The variables independent of time (sex, age group, and socioeconomic class) were stratified when entered in the Cox model (“stratified Cox regression,” [29]).

In an additional survival model, we used the number of mental disorders that occurred prior to the first SA as predictor, adjusting for sex, age cohort, socioeconomic class, and any prior separation event. Next, we computed the attributable fraction (AF) and the population attributable fraction (PAF). Here, the AF denotes the proportion of SAs that can be attributed to the predictor disorder within the exposed group. The PAF denotes the proportion of SAs that can be attributed to the predictor disorder within the total sample population. Therefore, both values represent the proportion of incident SAs that could conceivably be prevented if the respective mental disorder was prevented or treated effectively early on, among the exposed group (AF) and among the total sample (PAF). Both coefficients are interpreted under the assumption that the mental disorder is the cause of the subsequent SA. Interpretation also strongly depends on whether confounding factors as well as censoring have been taken into account [32]. We estimated the PAF, including its 95% confidence interval (95% CI), using the formula proposed by Natarajan et al. [33]: ${\text{Pe(HR}} - 1 )/{\text{HR}}$, where ${\text{Pe}}$ denotes the cumulative lifetime incidence of the temporally preceding DSM-IV mental disorder at T3 among those who subsequently attempted suicide. Missing AOO information in the presence of a reported disorder led to exclusion. *HR* is the hazard ratio coefficient from the Cox model. The AF formula is almost identical to the PAF formula, except that ${\text{Pe}}$ is not included: $\left( {{\text{HR}} - 1} \right)/{\text{HR}}$. The 95% CI for the PAFs was computed with the formulae $\left[ {{\text{PE}}_{L} \left( {{\text{HR}}_{L} - 1} \right)/{\text{HR}}_{L} ; {\text{PE}}_{U} \left( {{\text{HR}}_{U} - 1} \right)/{\text{HR}}_{U} } \right]$, where *L* and *U* denote the lower and upper bounds of the 97.5% CI, respectively. In computing the 95% CI for the AF, we adapted the formulae in the same way as in estimating the AF, that is, by excluding ${\text{Pe}}_{L}$ and ${\text{Pe}}_{U}$.

## Results

### Cumulative lifetime incidence and mean age of first onset of SAs

The cumulative lifetime incidence of SA at T3 was 5.5%. Estimates were higher for females (6.6%) than for males (4.4%; OR = 1.5, 95% CI = 1.08–2.18). The mean age at first SA was 16.7 years (95% CI = 15.7–17.6) and was comparable between males and females [*t*(167) = 0.67, *p* = 0.51]. There were 39 subjects who reported an SA but no age at the time of the first attempt. Therefore, these were omitted from any further analyses. For a general overview, see Table 1.

**Table 1:** Cumulative lifetime incidence and mean age of first suicide attempt in the completed 10-year Early Developmental Stages of Psychopathology Study

Sex	*N*	% wt	*N* SA	% wt	95% CI	Mean age (wt)	95% CI
Female	1488	51	106	6.6	5.2–8.0	16.4	15.3–17.4
Male	1533	49	63	4.4	3.2–5.6	17.0	15.4–18.7
Total	3021	100	169	5.5	4.6–6.4	16.7	15.7–17.6

*N* number of subjects, *wt* weighted, *SA* suicide attempt, *CI* confidence interval

Mean age based on *N* = 130 SA cases with age of first onset information

### DSM-IV disorders as risk factors for first SAs

**Fig. 1:**
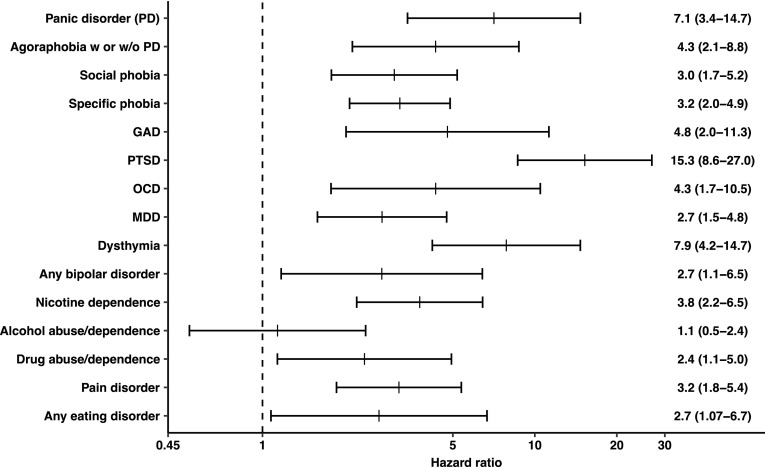
Temporal associations of prior DSM-IV mental disorder and the subsequent first lifetime suicide attempt, adjusted for sex, age cohort, socioeconomic status, any other prior mental disorder, and any prior separation event. Mean hazard ratios from a Cox regression model with time-dependent covariates are given with 95% confidence intervals in parentheses. The dashed vertical line represents a hazard ratio of 1.0, which means that the risk is elevated whenever the line is not crossed by the lower bound of the confidence interval. Log scaled transformation of horizontal axis. *PD* panic disorder, *GAD* generalized anxiety disorder, *PTSD* posttraumatic stress disorder, *OCD* obsessive–compulsive disorder, *MDD* major depressive disorder

Figure 1 displays the hazard ratio for each of the 15 DSM-IV mental disorders included in the analyses. All DSM-IV mental disorders except for any alcohol disorder elevated the risk for a first SA. Among anxiety disorders, PTSD showed the strongest association. Likewise, each of the affective disorders was positively associated with subsequent first SA, with dysthymia ranking first. The results for MDD and any bipolar disorder were similar to one another. Nicotine dependence showed an elevated risk for first SA as did any drug disorder. Finally, pain disorder and any eating disorder were also positively associated with subsequent first SA. The examination of the interactions between sex and prior mental disorders on predicting subjects’ first SA revealed no significant interaction effects.

**Fig. 2:**
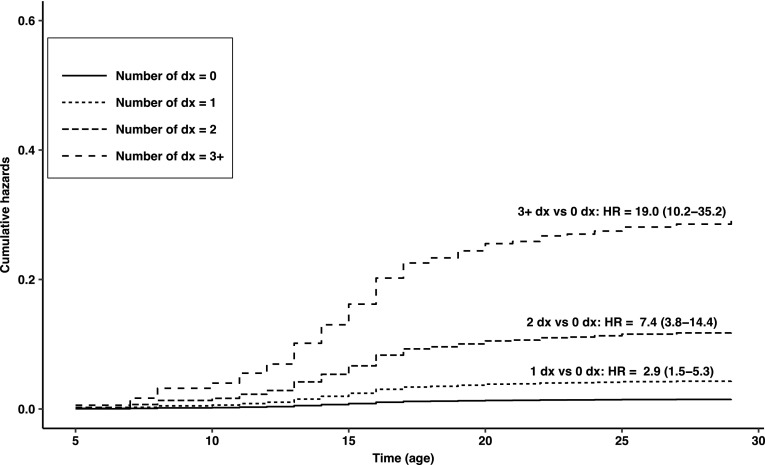
Cumulative hazard curves by number of DSM-IV mental disorders prior to the first lifetime suicide attempt, based on the reported age of onset and analyzed with the Cox regression model with time-dependent covariates. *Dx* disorders, *HR* hazard ratio; 95% confidence intervals are in parentheses

Figure 2 displays the burden of comorbidity with respect to the first onset of SA. Cumulative hazard curves show that the risk for first SA increased with increasing number of DSM-IV mental disorders: Hazard ratios were 2.9, 7.4, and 19.0 for 1 disorder, 2 disorders, and 3 disorders or more, respectively. We estimated that the risk for the subsequent first SA was elevated 8.9-fold on average (95% CI 5.7–13.8) with every unit increase in the comorbidity categories (i.e., 0, 1, 2, and 3 +).

### Attributable fractions

The PAF and AF estimates are shown in Table 2. Regarding the AF, within the exposed group, over 90% of first SAs could be attributed to PTSD, and 85% to panic disorder. GAD and OCD present AFs of 79 and 76%, respectively. Other disorders with notably high percentages were dysthymia (87%) and nicotine dependence (73%). Among the disorders that elevated the risk for the subsequent first SA, at a minimum drug abuse/dependence accounted for 57% of the latter. It is noteworthy that 81% of first SAs could be attributed to any DSM-IV disorder within the exposed group. Regarding the PAF, in the total sample, at least 11% of first SAs could be attributed to 7 of the 15 disorders. Highest values were found for specific phobia (31%), followed by nicotine dependence (22%). Further mental disorders with the potential to lower SA incidence by more than 11% were social phobia, dysthymia, PTSD, pain disorder, and MDD. On average, 63% of first SAs could be attributed to any DSM-IV mental disorder in the total sample of adolescents and young adults.

**Table 2:** Population attributable fraction (PAF) and attributable fraction (AF) for the impact of different mental disorders on subsequent first suicide attempt, adjusted for covariates, based on results of the Cox regression model with time-dependent covariates

DSM-IV mental disorder	*N*	AF	95% CI	PAF	95% CI
Panic disorder	2940	85.9	67.4–93.9	7.3	1.1–14.4
Agoraphobia w or w/o PD	2936	76.9	48.2–89.7	8.0	1.8–15.4
Social phobia	2938	67.2	39.7–82.2	14.8	4.9–26.0
Specific phobia	2920	68.7	49.0–80.8	31.2	16.8–45.6
GAD	2938	79.1	44.1–92.2	7.1	0.9–14.5
PTSD	2940	93.4	87.4–96.6	11.9	4.5–19.7
OCD	2938	76.9	36.3–91.6	4.7	0.2–10.5
MDD	2939	63.6	32.0–80.6	11.5	3.0–21.5
Dysthymia	2938	87.3	73.9–93.8	12.4	5.1–20.2
Any bipolar disorder	2940	63.5	3.5–86.3	4.8	−0.1–12.2
Nicotine dependence	2934	73.5	51.3–85.6	22.7	10.4–35.4
Alcohol abuse/dependence	2932	11.9	−106.4–62.5	1.5	−5.4–13.0
Drug abuse/dependence	2939	57.8	2.0–81.8	4.9	0.0–12.0
Pain disorder	2938	68.4	42.2–82.8	12.4	3.8–22.4
Any eating disorder	2938	62.6	−6.1–86.9	3.3	−0.1–8.9
Any DSM-IV disorder	2940	81.8	68.3–89.6	63.3	46.8–77.3

*N* number of subjects, *AF* attributable fraction, *PAF* population attributable fraction, *CI* confidence interval, *PD* panic disorder, *GAD* generalized anxiety disorder, *PTSD* posttraumatic stress disorder, *OCD* obsessive–compulsive disorder, *MDD* major depressive disorder

## Discussion

Several of our findings deserve to be highlighted: (1) In adjusted analyses, almost all temporally prior DSM-IV mental disorders were positively associated with the subsequent first SA. Highest elevated risks were found for PTSD (> 15-fold), dysthymia and panic disorder (> sevenfold), and GAD as well as OCD (each > fourfold). (2) Among the comorbidity categories of no prior mental disorder, 1, 2, and 3 or more prior disorders, on average each increasing unit was positively associated with the subsequent first SA (> eightfold). Risk elevations were threefold for 1 disorder, sevenfold for 2 disorders, and 19-fold for 3 or more disorders. (3) At the minimum, more than 50% of first SAs were preventable in the exposed group if the disorder had been causal and prevented. At the maximum, over 90% of first SAs were preventable in the PTSD group. (4) Half of all DSM-IV disorders each accounted for more than 11% of first SAs in the total sample. Total incidence reductions of first SA by more than 20% and even over 30% were possible, if nicotine dependence or specific phobia had been prevented, respectively.

Our results are best compared to those of the NCS-A study by Nock et al. [17] and the MAMHS study by Borges et al. [16]. They also used a representative sample of adolescents and young adults, included a wide range of specific DSM-IV mental disorders as risk factors for the subsequent first SA, and analyzed data with a discrete-time survival model based on retrospective AOO information. Our 5.5% overall cumulative lifetime incidence of SA is somewhat higher than the prevalence of 4.1% found by Nock et al. [17] and the prevalence of 3.1% found by Borges et al. [16]. A possible explanation for our higher estimate might be that our sample included older individuals up to age 34 and that we used prospective-longitudinal data for the estimation of the prevalence [34]. In good agreement with Nock et al. [17] and Borges et al. [16] as well as with our earlier analyses [10, 18], we observed a higher cumulative incidence of SA in women than in men.

In our study, PTSD strongly elevated the risk for a subsequent first SA. In the NCS-A data [17], PTSD was the only anxiety disorder with an elevated risk, whereas in the MAMHS data [16] PTSD was not predictive. When turning to representative community studies of adults, PTSD is also inconsistently reported to elevate the risk for (first onset) SA [for a review see 35]. In a recent community study of adults in South Africa, prior PTSD was found to be the strongest predictor for the subsequent first SA (adjusted for other DSM-IV mental disorders) [36]. In a cross-national analysis of representative adult samples from 21 countries around the globe, prior PTSD strongly elevated the risk for the subsequent first SA in both developed and developing countries [37], thus suggesting that ours was not a chance finding. Panic disorder was predictive in our study but not in either the NCS-A study [17] or the MAMHS study [16]. Among the anxiety disorders evaluated in the CHDS study [13], panic disorder elevated the risk for an SA in subjects 16–25 years old. Of the three anxiety disorders, it was the only one that remained significant across several sets of confounders, for example, other disorders and life stress. In a review on anxiety disorders and risk for suicide, Sareen [38] concluded that especially PTSD and panic disorder have often been found to be independent risk factors for SA, which is supported by our results for adolescents and young adults.

Among the affective disorders, the risk estimate of dysthymia was relatively large, compared to MDD and any bipolar disorder. In the MAMHS study [16], dysthymia had the highest risk estimate of all disorders analyzed. In the NCS-A data [17], the combined group of MDD/dysthymia elevated the risk for the first SA, yet the compound diagnosis prevents direct comparison to our results. In the CHDS data [12], affective disorders were reported as a compound diagnosis elevating the risk for an SA in 15–21 year olds, also preventing further comparisons to our results.

DSM-IV substance disorders performed relatively weakly in predicting the subsequent first onset of SA. In our study, drug abuse/dependence was predictive, yet alcohol abuse/dependence was not. In the NCS-A study [17], no substance disorder reached the significance level and in the MAMHS study [16] only drug abuse was predictive. However, nicotine dependence yielded a considerable risk estimate in our study, requiring an explanation. Aside from our own previous results where both non-dependent regular smokers and dependent smokers at baseline were at higher risk for their first future SA [15], we found one other study of young people that took the temporal sequence of tobacco use and SA into account. Using the MAMHS data of 12- to 17-year-old Mexicans, Miller et al. [39] reported elevated risks for the first SA along all different tobacco-related habits [i.e., (irregular) use, weekly use, daily use, dependence] and along all different sets of confounders. In the CHDS study, Boden et al. [40] reported associations between the number of cigarettes per day before the ages 16, 18, and 21 years and the subsequent (not first) SA between these ages. However, the temporal associations pooled over the 3 assessment periods attenuated to non-significance when controlled for non-observed fixed factors, suggesting that even more than 20 cigarettes per day was not an independent risk factor for subsequent SA. This contradiction may be explained by the smaller sample size (between 935 and 983) and the differences in the two data analyses.

Discussing our findings in light of results from adult population samples, all our significant associations have also been observed in adult samples [e.g., 37, 38, 41]. Our results extend these findings insofar as they impressively show that even in a young community sample, empirical evidence of temporal associations exists, adjusting for various confounders.

Several studies [42] have suggested that risk for subsequent SAs seems to be comparable across different mental disorders. Hoertel et al. [41], therefore, investigated in a large population sample whether this observed elevated risk for SA could be due to a general underlying psychopathology dimension. Using structural equation modeling, this group showed that effects of mental disorders on risk of SAs seem to occur through an underlying common general psychopathology dimension. On the other hand, Nock et al. [37], who also found associations between virtually all included mental disorders and subsequent SA in the World Health Organization World Mental Health Survey, demonstrated that the point risk estimates, vary remarkably by type of disorder. Given this variation in estimates, Nock et al. [37] interpreted their findings as arguing against an underlying common psychopathology dimension. Our results are in accordance with Nock et al.’s [37], since our risk estimates also differ remarkably across different disorders. Whether the associations between a variety of prior mental disorders and subsequent SAs are a result of a common psychopathological dimension is beyond the scope of our paper. This surely interesting question should be addressed in future analyses.

We also found that the number of DSM-IV mental disorders was a risk factor for the first SA in our study. Having 1, 2, or 3 or more mental disorders all elevated the risk for a subsequent first SA. This finding is in relatively good agreement with other community surveys [e.g., 43, 44]. Comparable to earlier EDSP analyses based on cross-sectional baseline data [10], our data even support a clear dose–response relationship between number of comorbid mental disorders and the first SA.

AFs have not been estimated so far in studies evaluating the risk of mental disorders for the first SA. It certainly seems impressive that any of the mental disorders that elevated the risk for the subsequent first SA bear the potential of reducing this tragic outcome by at least 57% (drug abuse/dependence) and up to 93% (PTSD). Moreover, anxiety disorders seem to be a very important intervention target in the subpopulation of adolescents and young adults, because not only is the AOO of anxiety disorders earlier compared to other groups of mental disorders but also SA might be prevented to a considerable extent.

PAFs in conjunction with our study characteristics were reported in the CHDS study [13]. Anxiety disorders accounted for 7.5% of SAs in the total birth cohort sample of individuals aged 16–25 years. In our study, PAFs for single anxiety disorders ranged from 4.7 (OCD) to over 30% (specific phobia). Unfortunately, the results of the CHDS study [13] and the results of our study cannot be compared directly due to the use of the disorder group (anxiety) instead of single anxiety disorders in the former. When turning to adult community samples that reported associations between prior mental disorders and the subsequent first SA we found only one study that reported PAFs. Bernal et al. [45] reported that MDD accounted for 28% of first SAs in a sample of more than 21,000 adults (aged 18 years and over) across 6 European countries. GAD accounted for 4% of first SAs. The PAF of 28% is almost 3 times as much as our result of 11.5% for MDD. Most likely this is due to differences in age and other methodological characteristics. In other studies with adult community samples, PAFs were reported not for single disorders but for disorder groups only (e.g., mood disorders, any mental disorder; [46–48]), were based on results that did not take into account the temporal order of risk factor and outcome [49–51], or did not point to the subsequent first SA [41].

Our study points out how essential it is to consider the context of SAs, in terms of age, sex, and psychopathology. When considering SAs or suicidal behavior in general, heuristics do more harm than good; that is, inquiring about suicidal phenomena only if the (young) patient reports depressive symptoms is important, yet not inquiring otherwise might often be fatal. Furthermore, a relatively rare phenomenon such as suicide and all of its cognitive and behavioral derivatives should be approached on the population level with much more determination, as discussed by Knox et al. [52]. The determination to prevent SAs on the population level of course is synonymous with major efforts professionally, politically, and monetarily. Our study might serve to guide the selection of promising prevention targets for the general population of adolescents and young adults. After all, “it seems likely that earlier identification and earlier symptomatic relief is an important component of the prevention and treatment of youth suicidal behavior” [53].

The present study has several methodological strengths. First, we used a representative community sample of adolescents and young adults with an observation period that fully included the high-risk period of both the first onset of mental disorders and the first SA. Second, our analyses are strengthened by the inclusion of all individuals who reported an SA rather than individuals who received medical attention, and by the inclusion of a comprehensive set of predictor diagnoses that allowed us to evaluate a broad range of mental disorders. Third, we used an interviewer-administered standardized interview.

Our study also has several limitations. First, our analyses were based on self-reported data, which are always prone to recall bias. However, this bias may have been lessened by our longitudinal study design of 3 follow-up assessments over a 5- to 10-year period, which decreased the time frame for retrospective assessments. Second, several disorders known to be associated with suicidal behavior were not analyzed in our study (e.g., schizophrenia, personality disorders), because they were not assessed. Third, the assessment of SA at T0 and T1 was limited to individuals who reported depressive symptoms over at least 2 weeks. Although this might have led to a more conservative prevalence estimate initially, the later follow-up waves assessed lifetime SA in all subjects. Fourth, the AFs and PAFs are preliminary quantitative appraisals of the impact of mental disorders on the risk for onset of a first SA. The preliminary status and the inherent assumption of causality warrant caution in interpreting the results.

These limitations notwithstanding, our study provides valuable new information about mental disorders and SA in adolescence and young adulthood. From a public health perspective, our AFs suggest that the prevention of mental disorders among adolescents and young adults could substantially reduce the incidence of SAs. Our results also clearly demonstrate the importance of considering not only depression but also the full range of mental disorders when evaluating the risk for suicidal behavior. Given the strong associations between comorbidity and SA, clinicians should always conduct a suicide risk assessment among patients presenting with multiple mental disorders. Finally, our results point to the need for future work to increase our understanding of the increased risk for suicidal behaviors during adolescence and of the causal pathways linking mental disorders to suicidal behaviors.
